# Active microbiota persist in dry permafrost and active layer from Elephant Head, Antarctica

**DOI:** 10.1093/ismeco/ycad002

**Published:** 2024-01-10

**Authors:** Claudia Wood, Alyssa Bruinink, Elizabeth Trembath-Reichert, Mary Beth Wilhelm, Chanel Vidal, Edward Balaban, Christopher P McKay, Robert Swan, Barney Swan, Jackie Goordial

**Affiliations:** School of Environmental Sciences, University of Guelph, 50 Stone Rd E, Guelph, Ontario N1G 2W1, Canada; School of Environmental Sciences, University of Guelph, 50 Stone Rd E, Guelph, Ontario N1G 2W1, Canada; School of Earth and Space Exploration, Arizona State University, 781 Terrace Mall, Tempe, AZ 85287, United States; Space Science & Astrobiology Division, NASA Ames Research Center, Moffett Field, CA 94035, United States; School of Earth and Space Exploration, Arizona State University, 781 Terrace Mall, Tempe, AZ 85287, United States; Space Science & Astrobiology Division, NASA Ames Research Center, Moffett Field, CA 94035, United States; Space Science & Astrobiology Division, NASA Ames Research Center, Moffett Field, CA 94035, United States; 2041 Foundation, 130 Wescott Ct, Auburn, CA 95603, United States; 2041 Foundation, 130 Wescott Ct, Auburn, CA 95603, United States; School of Environmental Sciences, University of Guelph, 50 Stone Rd E, Guelph, Ontario N1G 2W1, Canada

**Keywords:** dry permafrost, microbial activity, radiorespiration, cryophile, polar microbiology

## Abstract

Dry permafrost is a challenging environment for microbial life due to cold, dry, and often oligotrophic conditions. In 2016, Elephant Head, Antarctica, was confirmed as the second site on Earth to contain dry permafrost. It is geographically distinct from the McMurdo Dry Valleys where dry permafrost has been studied previously. Here, we present the first study of the microbial activity, diversity, and functional potential of Elephant Head dry permafrost. Microbial activity was measured using radiorespiration assays with radiolabeled acetate as a carbon source at 5, 0, and −5°C. Low, but detectable, rates of microbial activity were measured in some samples at 0 and −5°C. This is distinct from previous studies of McMurdo Dry Valley dry permafrost which concluded that dry permafrost represents a cold-arid limit to life on the planet. The isolation of cold-adapted organisms from these soils, including one capable of subzero growth, further supports that the Elephant Head dry active layer and dry permafrost harbor viable microbial life, which may be active *in situ*. Metagenomic, 16S rRNA gene, and internal transcribed spacer and amplicon sequencing identified similar microbial communities to other Antarctic and cold environments. The Elephant Head microbial community appears to be adapted for survival in cold, dry, and oligotrophic conditions based on the presence of cold adaptation and stress response genes in the metagenomes. Together, our results show that dry permafrost environments do not exclude active microbial life at subzero temperatures, suggesting that the cold, dry soils of Mars may also not be as inhospitable as previously thought.

## Introduction

Dry permafrost is ground that remains below 0°C throughout the year and has negligible water content [1]. Survival in dry permafrost is challenging, and life is limited to microorganisms that can tolerate persistently subzero, dry, and typically oligotrophic conditions. The occurrence of dry permafrost on Earth is rare, limited to only two confirmed locations in Antarctica so far [1-3]. Until recently, the microbiology of dry permafrost had only been studied in the high-elevation Stable Upland Zone of the McMurdo Dry Valleys (MDVs) in the Transantarctic mountains of Southern Victoria Land, Antarctica [4, 5]. Previous studies of dry permafrost from a high elevation MDV, University Valley, revealed the presence of viable microbial life but were unable to detect activity at environmentally relevant temperatures and concluded that a natural limit to active life may be reached in dry permafrost [4, 5]. Viable cells were confirmed through the isolation of bacteria and fungi, though the number of culturable organisms was limited (6 isolates) compared to the number typically obtained from other permafrost soils globally (60–158 isolates) [6-8]. Dry permafrost and other cold-dry or hyper-arid soils from the Antarctic and Atacama Desert commonly have low amounts of microbial biomass, with cell counts that typically range from 10^2^ to 10^6^ cells/g of soil [5, 9, 10]. Despite low microbial biomass, dry permafrost in University Valley contains high microbial diversity, and the microbiota possess genomic adaptations for survival in the cold, dry, and oligotrophic conditions [5]. Studies of cold and ice-free soils from elsewhere in Antarctica have also measured low rates of activity and amounts of extractible DNA that decrease to nondetectable levels with increased elevation (corresponding to colder and drier soils) [9]. These results suggest that these conditions may be prohibitive to microbial life [9].

In late 2016, dry permafrost was confirmed at a second location on Earth, in Elephant Head, Ellsworth Land, Antarctica [1, 2]. Elephant Head is located ~2100 km away from the MDVs and provides the first geographically distinct site to compare with the microbial ecology of dry permafrost to the MDVs. Though dry permafrost is present at Elephant Head, temperature data indicate that it is slightly warmer and has higher relative humidity above the ice table than University Valley [1], therefore soils here may be more hospitable to life.

This study presents the first observations of microbial diversity and activity of dry permafrost collected from Elephant Head, Antarctica. These are the first microbial activity measurements from dry permafrost outside of the MDVs, providing a second reference point for defining habitability and the limits of active life in cold-arid environments on Earth. In both University Valley and Elephant Head, dry permafrost overlies ice-cemented ground [1, 11] similar to that observed by the Mars Phoenix lander [12], making rare dry permafrost sites on Earth highly relevant analogs for investigating the habitability of the north polar region of Mars.

## Materials and Methods

### Site description, field, and lab sampling approaches

**Figure 1:**
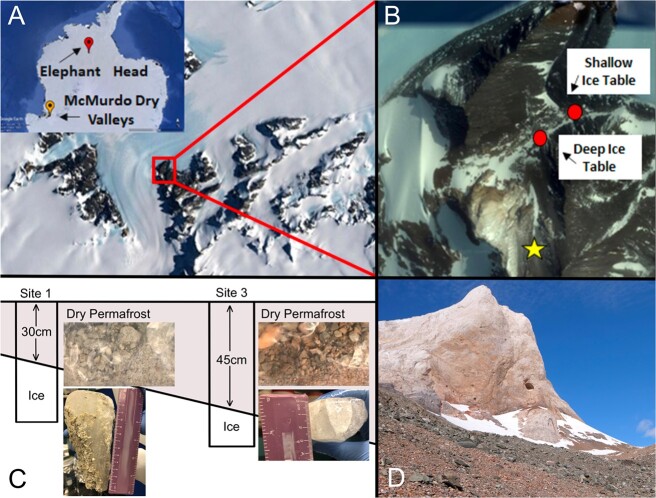
(A) Location of Elephant Head, Antarctica; (B) shallow ice table (Site 1), deep ice table (Site 3); sites are 0.3 km apart; star indicates the location of Elephant Head rock formation; (C) dry permafrost and ice table depths at Sites 1 and 3; photos show dry permafrost and ice cores that were collected from each site; (D) Elephant Head rock formation.

Elephant Head is located in Ellsworth Land, Antarctica (79°49.213′S, 83°18.860′W, 718 m) as described in McKay *et al*. Site naming conventions are kept the same in this study as in McKay *et al*.; the location of Site 1 is 79°49.106′S,83°18.139 W and Site 3 is 79°49.211S, 83°18.835 W (Fig. 1). Site 1 and Site 3 are 0.3 km apart. The average summer atmospheric temperature in Elephant Head is −10.3°C, with a yearly average of −20.3°C [1] (Supplemental Table 1). Surface soils at Site 3 warmed above 0°C for 490 h throughout the summer, and the depth to dry permafrost estimated to be at 13 cm [1]. No long-term data are available for Site 1. There is no compositional change associated with surface samples compared to dry permafrost at this site, as there is no water to thaw from ice to liquid [1]. However, we will refer to samples from the 0–10 cm depth as dry “active” layer based on temperatures exceeding 0°C. Samples were collected in January 2020 as part of a 2041 Foundation Antarctic Expedition. Samples of dry permafrost were collected aseptically using sterile disposable sampling spoons in 10 cm increments. The ice table underlying dry permafrost was located at 30 cm and 45 cm depth at Sites 1 and 3, respectively. Ground ice was sampled using a handheld battery powered drill and 3.5 inch diameter dry/wet diamond core drill bit (Concord Blades). A 13 cm long core of ground-ice was recovered from Site 1, and a 3 cm long core of ground-ice from Site 3. Samples were kept at −20°C in the field, during transport, and stored at −20°C in the lab until processing.

Dry permafrost and ice core subsamples were aliquoted at room temperature (~25°C) in a biological safety cabinet with sterile equipment. For ice cores, the outer 0.5 cm was first removed by scraping with a sterile metal scoopula, then the core was submerged briefly (~1 s) in 10% bleach followed by a rinse with prechilled sterile water.

### Permafrost radiorespiration assays

Microcosms were set up as in Goordial *et al*. [5]. Dry active layer/permafrost (5 g) or ice (5 ml thawed volume) was added to 20 ml serum vials. Microcosms were set up in triplicate for each sample with triplicate negative controls (autoclaved twice for 1.5 h at 120°C, with 24 h between autoclaving). Microcosms were spiked with 0.043 μCi (~95 000 disintegrations per minute, dpm) of 1,2-^14^C acetic acid in a 20 μl volume. An additional 20 μl of unlabeled acetic acid (3.75 M) was also added per microcosm. In total, 40 μl of 15 mM acetate was added to each microcosm vial. The CO_2_ traps consisted of 0.5 ml of 1 M KOH in a glass tube placed inside of the serum vial. Positive controls were set up as above, using permafrost from the Northwest Territories, Canada (68°18.8514 N, 133°42.852 W), later referred to as “Arctic.” Microcosms were incubated at 5, 0, or −5°C for 318 days. For each time point measurement, 0.5 ml of KOH was removed from the trap, the trap was rinsed with an additional 0.5 ml KOH, and the trap contents and rinse were added to a scintillation vial with 18 ml of ScintiVerse™ BD Cocktail (Fisher Chemical). The KOH trap was immediately refilled with 0.5 ml of KOH to continue incubations. Measurements of radioactivity (dpm) were determined by liquid scintillation spectrometry with a 3-min count time on a Beckman Coulter (CA, USA) LS 6000SC, and the percent mineralized fraction was calculated. A paired one-tailed *t*-test was used to compare the means of each site and depth to its corresponding negative control at Day 318.

### Cultivation and characterization of isolates

Dry active layer/permafrost (0.5 g) was added to 1.5 ml of liquid enrichment media and incubated at 5 or 15°C for 10 weeks aerobically. Liquid enrichment media used were: 0.1% sodium pyrophosphate, 0.1% sodium pyrophosphate +2% sodium chloride (NaCl), 1/10 strength Reasoner’s 2A broth (R2B) media, 1/10 strength R2B + 5% NaCl, R2B, or R2B + 5% NaCl. Subsections used for cultivation were 0–10 cm (Sites 1 and 3) and 30–40 cm depth from Site 3. After 1, 3, 5, and 10 weeks of incubation, 100 μl of the liquid-enriched sample was plated onto solid media. Each liquid enrichment culture was plated onto the same solid media, and in addition, onto all previously listed media with a higher carbon content.

Colonies were selected for isolation based on distinct visible properties (size, color, texture; 45 colonies). Colonies were streaked for isolation and those that were isolated with enough biomass were sequenced (21/45). To identify isolates, the 16S rRNA gene was amplified using PCR with primers (27F-5′-AGAGTTTGATCCTGGCTCAG-3′ and 1492R-5′-TACGGYTACCTTGTTACGACTT-3′). PCR consisted of an initial denaturation for 5 min at 95°C followed by denaturation (30 s, 94°C), annealing (40 s, 50°C), and extension (1 min 40 s, 72°C) for 35 cycles plus a final extension phase at 72°C for 10 min. Sanger sequencing occurred at the TCAG sequencing center, Toronto, Canada. Isolates were identified using the Classifier tool of the Ribosomal Database Project (RDPII) [13] and the NCBI GenBank database. Those that were able to regrow to high enough biomass (13/21 which were identified) were characterized on 11 different types of media to determine optimal growth conditions and tolerance to salt and glycerol, which are required media freezing point depressants required to test subzero temperature growth. Isolates were tested for growth at 37 to −5°C and on media: R2A, 1/2 R2A, 1/10 R2A, R2A + 5% NaCl, 1/10 R2A + 5% NaCl, 1/2 R2A + 8% NaCl, R2A + 3% glycerol, ½ R2A + 3% glycerol, 10 R2A + 3% glycerol, 1/10 TSA.

### Nucleic acid sequencing

DNA was extracted using the Qiagen Powerlyzer Powersoil Kit with a modified procedure for low biomass samples as in Goordial *et al*. [5]. Briefly, DNA was extracted from 10 g of permafrost total, aliquoting 1 g per extraction tube (ten extractions per sample). Kit glass beads were removed from extraction tubes prior to the addition of permafrost. One sample (e.g. Site 1 0–10 cm depth) was processed per day, including a negative control with every extraction procedure. A bead-beating step was added to the manufacturer’s protocol to help with cell lysis using a FastPrep FP120. DNA from the 10 extractions was pooled onto one DNA binding column and eluted using 50 μl of water. Negative controls were included at each step using water and kit glass beads instead of permafrost.

DNA concentration was measured using a Qubit with the HS dsDNA assay kit. Low (0.0662 ng/μl) to below detection limit concentrations of DNA were measured. PCR amplification directly from DNA extractions failed to produce PCR products. To amplify DNA for sequencing, multi-displacement amplification was performed using the Repli-g Midi Kit (Qiagen) according to the manufacturer’s protocol with 2.5 μl of input DNA. For Site 3 sample 0–10 cm depth, the Repli-g single cell kit (Qiagen) had the highest DNA yield and was used for downstream analysis. PCR amplification of (whole genome amplified) DNA was carried out targeting the 16S rRNA gene (515F-5′-GTGYCAGCMGCCGCGGTAA-3′ and 926R-5′-CCGYCAATTYMTTTRAGTTT-3′) [14, 15]. PCR conditions were as described for the isolates. Samples with a visible PCR product band (and their corresponding negative controls) were used for downstream sequencing.

Library preparation and DNA sequencing occurred at the Integrated Microbiome Resource (Dalhousie University, Halifax, Nova Scotia, Canada). Amplicon sequencing was performed using Illumina MiSeq with primers targeting the V4V5 region of the 16S rRNA gene (515F, and 926R) [14, 15], and the ITS2 region in fungi (ITS86(F) = GTGAATCATCGAATCTTTGAA, ITS4(R) = TCCTCCGCTTATTGATATGC) [16]. Metagenomic library preparation was performed using the Illumina Nextera Flex kit and sequenced using the Illumina NextSeq.

### Sequencing analysis

#### 16S rRNA gene and internal transcribed spacer amplicon data

Amplicon sequences were processed using DADA2 (v 1.22.0) [17] in R studio. Bacterial taxonomy was assigned using the SILVA database (v 138.1) [18, 19]. Fungal taxonomy was assigned using the UNITE internal transcribed spacer (ITS) database (v 8.3) [20]. In addition to quality control and chimera detection from the DADA2 pipeline, sequences were manually screened for potential contaminants (Supplemental Methods—Manual Contaminant screening of amplicon sequence data; Supplemental Figure 1).

#### Metagenomic data

Metagenomic sequences were normalized using BBNORM from the BBTools suite (v 38.18) (https://sourceforge.net/projects/bbmap/files/) using target = 30 min = 3 kmer = 21. Sequences were trimmed using Trimmomatic (v 0.39) with the default settings (LEADING:3 TRAILING:3 SLIDINGWINDOW:4:15 MINLEN:36) [21]. Reads were co-assembled using SPAdes (v 3.13.0) [22] and mapped using BBMAP from BBTools. Reads were binned with Metabat2 (v 2.15) with a minimum contig length of 1500 [23]. Metagenome-assembled genomes (MAGs) were imported into Anvio (v 7.1) using anvi-import-collection [24, 25]. Completion and redundancy of MAGs were evaluated using CheckM (v 1.1.3) (https://github.com/Ecogenomics/CheckM). MAGs were investigated using anvi-interactive and manually refined using anvi-refine based on sequence composition and differential coverage. Anvi-refine was not able to reduce contamination in some MAGs (3 and 5) based on sequence composition, potentially due to the presence of multiple closely related strains. MAGs were identified using the Genome Taxonomy Database Toolkit (v 2.1.0) with database release 207 [26]. No reads were recruited from the negative control for any of the MAGs produced and reads from the negative control were removed from the unbinned section (those sequences that could not be assigned to a bin) before annotation. Genomes were annotated using Prokka (v 1.14.6) [27] and GhostKoala [28]. Completeness of metabolic pathways was visualized with KEGG Decoder [29]. The presence of trace gas metabolism genes was evaluated using the BLASTx function of DIAMOND (v 2.0.15) [30]. Prokka-annotated DNA sequences were searched against protein sequences from the Greening lab metabolic marker gene database (v 3) (Leung & Greening 2020) [31]. As in Dragone *et al*. and Bay *et al*. [32, 33], a minimum query coverage of 80%, a minimum percent identity of either 60% (PmoA, MmoX, CoxL) or 50% (NiFe), and a maximum e-value threshold of 10^−10^ were used to filter matches.

### Permafrost geochemistry, fatty acid methyl ester analysis, and direct microscopic cell counts

Anion analysis, elemental analysis, and fatty acid extraction were performed on samples as described in Supplemental Methods (Supplemental Methods—Anion analysis, Elemental analysis, Fatty acid analysis). Gravimetric moisture content was measured by weighing soil before and after baking at 110°C for 30 h. Microscopic cell counts were performed on soil from Site 3 0–10 cm and Arctic permafrost, as described in Supplemental Methods (Supplemental Methods—Direct microscopic cell counts).

## Results and Discussion

### Elephant Head soils are oligotrophic and low in microbial biomass

Total carbon content ranged from 5 to 11 wt% C across sites. Site 1 (5–8 wt% C) had slightly lower carbon content than Site 3 (10–11 wt% C, Supplemental Table 2). This carbon was assumed to be predominantly carbonate, which was confirmed by acid digestion of the shallowest horizon from Site 3. The resulting total organic carbon content in Site 3 0–10 cm was 0.07 wt% with a significantly more depleted carbon isotope composition consistent with a biological origin (−28‰ δ13C). The undigested carbon isotope values were between 0.5 and 1‰ δ13C, consistent with carbonate composition. The total carbon content in Elephant Head samples was higher compared to University Valley (0.01–0.05 wt% total C) due to the presence of carbonate [5]. Nitrogen content (0.004–0.011 wt%), however, was consistent with those in University Valley [5]. Nitrogen isotope compositions ranged between −10 and −20‰ δ15N, with slightly more depleted values at Site 3 compared to Site 1. Dominant anions chloride and nitrate increased with depth at both sites (Supplemental Table 3). However, the range in concentrations differed between sites, with values differing across an order of magnitude at Site 3, but not Site 1. Increasing chloride ion concentrations may indicate that prior snowmelt occurred and flushed ions into the top ~10 cm of soils at Site 3. Sulfate followed a similar increasing with depth trend for Site 3, but not for Site 1, which had almost twice the concentration at the top horizon (428 mg/kg) compared to the two deeper horizons (<280 mg/kg). Ice concentrations were much lower (5–1 mg/kg) for all anions and consistent between sites.

Site 3 0–10 cm contained low amounts of microbial biomass, 3.31 × 104 cells/g of dry permafrost (Supplemental Table 2). Site 3 0–10 cm had the only measurable amount of DNA (0.0662 ng/μl) prior to whole genome amplification, suggesting that this sample represents the upper amount of biomass present across the samples (Supplemental Table 2). The cell density is one order of magnitude higher than what was measured in University Valley dry permafrost (10^3^ cells/g), but much lower than the Arctic permafrost positive control which contained 1.12 × 10^8^ cells/g of soil. It is also lower than the cell counts found in the lower elevation MDVs (10^7^–10^9^ cells/g) [33-36] or from Canadian Arctic permafrost samples (10^5^–10^8^ cells/g) [7, 34]. Lipid extractions yielded only six unique fatty acid methyl esters (FAMEs) at Site 3 that were only slightly above the limit of detection using 110 g of soil and techniques tailored to hyperarid soils (Supplemental Methods—Fatty Acid Analysis). The low biomass observed is in line with measurements of cell counts and lipid concentrations in hyperarid surface soils in the Atacama Desert 10^3^–10^5^ cells/cm^3^ [10, 38, 39].

### Microbial activity in Elephant Head dry active layer/permafrost

**Figure 2:**
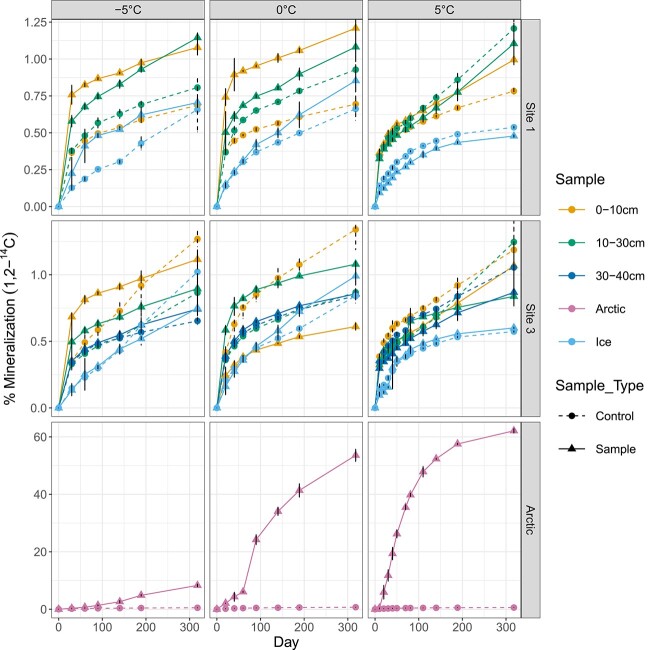
Microbial activity measured through the mineralization of radiolabeled acetate (1,2-14C) to carbon dioxide (CO_2_) in Elephant Head dry permafrost, Elephant Head ice samples, and Arctic permafrost (positive control); measurements are cumulative; dashed lines show heat killed negative controls for each sample; error bars show standard deviation of triplicates.

In general, microbial activity in Elephant Head samples was low compared to Arctic permafrost, reaching a maximum of 1.25% and 60% acetate mineralization, respectively (Figure 2). Levels of mineralization in dry active layer, dry permafrost, and ice samples were close to what was seen in negative controls from abiotic release (including Arctic negative controls). Three samples of dry permafrost (Site 1 10–30 cm 0°C, Site 3 10–30 cm 0°C, Site 1 10–30 cm −5°C) had significantly higher cumulative mineralization at Day 318 compared to their corresponding negative controls (Supplemental Table 4). All dry permafrost and ground ice samples incubated at 0 and −5°C from Site 1 had elevated acetate mineralization compared to the background (0.04%–0.5% relative increase), but not all were significant (Supplemental Table 4). For Site 3, most samples were elevated compared to their corresponding background levels for the first ~150 days of incubation; however, by Day 318, this activity was difficult to distinguish or was lower than background levels of corresponding negative controls. The ability to detect even low amounts of activity in Elephant Head dry permafrost at −5°C is notable, as previous work using the same methods on University Valley dry permafrost was unable to detect activity below 5°C [5]. The difference in activity level may be attributable to differences in regional climactic conditions as Elephant Head is slightly warmer than University Valley (average air temperature −20.3 vs. −23.4°C) and has higher relative humidity (average air RH 68.7% vs. 45.4%) (Supplemental Table 1). Although only slightly warmer and wetter, these conditions would be more favorable for microbial life [1, 5].

Incubation temperature did not correlate with microbial activity in any of the Elephant Head samples. Warmer temperatures are typically associated with higher microbial activity, as cellular processes occur faster due to kinetics and less stress on cellular processes. This expected temperature dependence was observed in the Arctic permafrost positive controls, where microcosms were 95% and 8.7% mineralized after 318 days at 5 and −5°C, respectively.

Some sites and depths had levels of abiotic activity in the negative control that were higher than the samples after Day 318. The use of an alternative labeled substrate such as glucose may help to reduce background levels of production; however, glucose was not used as it may inhibit growth of cells that are used to oligotrophic conditions [40], and due to the limited available sample volume for activity assays.

### Cultivation of cold-adapted organisms

Cultivation yielded 21 isolates and included members of the genera *Arthrobacter*, *Actinobacterium*, *Hymenobacter*, *Kocuria*, *Rhodococcus*, and *Sphingomonas* (Table 1). These genera have frequently been isolated from other Antarctic permafrost studies [5, 9, 40-42]. All isolates grew at 0°C (except 1, *Pseudarthrobacter*), indicating that these organisms are adapted for growth under cold conditions. Most isolates were pigmented (20/21) and were either pink, orange, or yellow. Pigmentation is known to protect against a variety of stressors including oxidative stress, solar radiation, and cold temperatures [43-45]. Of the 13 isolates tested for growth, 10 were able to tolerate salt concentrations of at least 5% (Table 1). One *Kocuria* sp. isolate was able to tolerate 12% salt. Increased salt and osmotolerance are important for survival in subzero conditions, where liquid water may only exist in niches where increased solutes depress the freezing point of water [47]. Most isolates were related to organisms in the GenBank database from other cold environments globally, including many Antarctic regions (Table 1). *Arthrobacter* was the most frequently isolated species (seven isolates), one of the *Arthrobacter* isolates displayed cell division at −5°C. Cultivation attempts on Elephant Head dry permafrost yielded more isolates than previous attempts on University Valley dry permafrost (only six isolates, one of which was capable of subzero growth) [48, 49]. The ability to cultivate a variety of psychrotrophic isolates including one capable of subzero growth supports that this environment harbors viable microbial life which may be active *in situ.*

**Table 1:** Characteristics of cultured isolates from Elephant Head dry permafrost.

**Strain Name**	**Isolate**	**Colony morphology**	**Environment of closest BLAST match**	**% Similarity closest BLAST match**	**Sample isolated from**	**Temp growth range (°C)**	**Salinity growth range (%)**	**Isolation media**	**Isolation temp (°C)**	**GenBank Accession 16S rRNA gene**
Ant-EH-1	*Arthrobacter agilis*	Pink, small, circular, smooth edges, opaque	Cheese from cows milk (NR_170400)	99.13	Site 1, 0–10 cm	−5 to 30	0–8	R2A	15	OQ383637
Ant-EH-2	Unidentified *Microbacteriaceae*	Cream, microcolonies, circular, entire	Soil, Darwin Mountains, Antarctica (KC442690)	98.92	Site 3, 0–10 cm	0–25	0–5	1/10 R2A	15	OQ383638
Ant-EH-3	*Arthrobacter* sp.	Light pink, round, opaque, smooth edges	Soil, Sor Rondane Mountains, Antarctica (KY386623)	98.35	Site 1, 0–10 cm	ND	ND	R2A + 5% NaCl	15	OQ383889
Ant-EH-4	*Pseudomonas* sp.	Light pink, round, translucent edges	Fresh white button mushroom (MH235992)	99.76	Site 1, 0–10 cm	ND	ND	R2A	15	OQ383890
Ant-EH-5	*Arthrobacter* sp.	Pink, round, smooth edges, opaque	Soil, La Gorce Mountains, Antarctica (DQ351727)	98.52	Site 1, 0–10 cm	0–25	0–5	R2A	15	OQ383891
Ant-EH-6	*Williamsia* sp.	Pink, round, opaque, smooth edges	Skin (MK465379)	99.21	Site 1, 0–10 cm	ND	ND	1/2 R2A	5	OQ383892
Ant-EH-7	*Hymenobacter* sp.	Pink, round, smooth edges, translucent edges	Soil, Sor Rondane Mountains, Antarctica, (KY386591)	96.82	Site 3, 0–10 cm	0–25	0	1/10 R2A	15	OQ383639
Ant-EH-8	*Kocuria* sp.	Pink, opaque, circular	Soil, cold desert, Indian Himalayas (MZ314095)	99.29	Site 1, 0–10 cm	0–37	0–12	1/2 R2A + 5% NaCl	15	OQ383640
Ant-EH-9	*Pseudoarthrobacter* sp.	White, large, tear shaped, smooth edges, opaque	Rhizosphere soil, China (MT318454)	99.4	Site 3, 30–40 cm	0–30	0–5	1/10 R2A	5	OQ383893
Ant-EH-10	*Arthrobacter* sp.	Pink, circular, smooth edges, slightly raised	Soil, Darwin Mountains, Antarctica (KC442549)	96.7	Site 3, 0–10 cm	0–15	0–5	R2A	15	OQ383894
Ant-EH-11	*Arthrobacter* sp.	Bright pink, irregular shape, and edges	Soil, La Gorce Mountains, Antarctica (DQ351727)	99.35	Site 1, 0–10 cm	0–25	0–5	1/2 R2A	15	OQ383895
Ant-EH-12	*Arthrobacter* sp.	Pink, circular, smooth edges	Soil, Sor Rondane Mountains, Antarctica, (KY386623)	98.29	Site 1, 0–10 cm	ND	ND	1/2 R2A	15	OQ383896
Ant-EH-13	*Microlunatus* sp.	Yellow, circular, smooth edges, translucent	Soil, Darwin Mountains, Antarctica (KC442616)	99.73	Site 1, 0–10 cm	ND	ND	TSA	15	OQ383897
Ant-EH-14	Unidentified *Nocardiaceae*	Light pink, irregular shape and edges	Soil, La Gorce Mountains, Antarctica (DQ351736)	100	Site 3, 30–40 cm	ND	ND	1/2 R2A	15	OQ383898
Ant-EH-15	*Hymenobacter* sp.	Bright pink, raised and textured, opaque	Soil, Arctic (MT068548)	98.59	Site 3, 30–40 cm	0–25	0–5	TSA	15	OQ383899
Ant-EH-16	*Microbacterium* sp.	Orange, circular, opaque	Soybean stem, Japan (AB461207)	99.12	Site 3, 30–40 cm	0–37	0–8	TSA	15	OQ383900
Ant-EH-17	*Sphingomonas* sp.	Orangish-pink, circular, smooth edges, raised	Volcanic deposits, Hawaii (DQ490369)	98.9	Site 3, 0–10 cm	0–30	0	1/2 R2A	5	OQ383901
Ant-EH-18	Unidentified *Microbacteriaceae*	Yellow, small, circular, smooth edges, opaque	Soil, Darwin Mountains, Antarctica (KC442690)	98.11	Site 3, 0–10 cm	ND	ND	1/2 R2A	5	OQ383902
Ant-EH-29	*Microlunatus* sp.	Light orange, irregular edges, bumpy texture, opaque	Soil, Darwin Mountains, Antarctica (KC442612)	98.11	Site 1, 0–10 cm	ND	ND	TSA	15	OQ383903
Ant-EH-20	*Arthrobacter* sp.	Bright pink, punctiform, opaque, smooth edges	Soil, Darwin Mountains, Antarctica (KC442686)	96.04	Site 1, 0–10 cm	0–25	0–5	1/2 R2A	15	OQ383904
Ant-EH-21	*Hymenobacter* sp.	Light pink, smooth translucent edges, opaque middle	Grass soil, South Korea (KY412788)	86.72	Site 1, 0–10 cm	0–25	0	R2A	15	OQ383905

### 16S rRNA gene, internal transcribed spacer and metagenome-assembled genome microbial diversity

**Figure 3:**
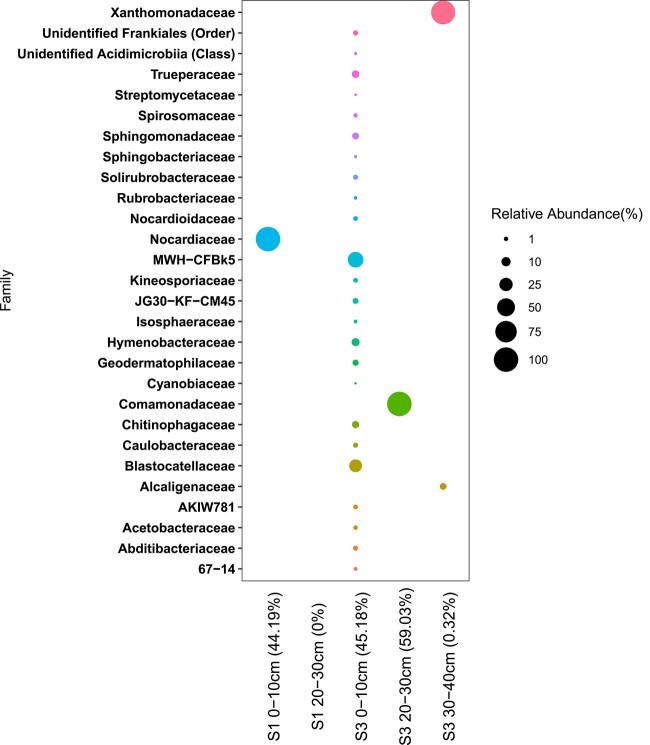
16S rRNA gene amplicon sequences following removal of sequences classified as contaminants or likely contaminants based on presence in negative controls and GenBank environment match; percent indicates the percentage of sequences that were retained; negative controls are not shown.

A total of ~190 000 16S rRNA gene sequences and ~11 000 ITS sequences were obtained before processing (Supplemental Tables 5 and 6). Sequencing of the 16S rRNA gene (227 ASVs) and 11 MAGs revealed that the bacterial community consisted of microbiota which have been previously identified in cold and arid regions of the Antarctic (Fig. 3) [5, 49-51]). MAGs were largely composed of reads from Site 3 0–10 cm with very few reads recruited from any other sample. MAG completion ranged from 0% to 96% (based on the presence of single copy genes) and included five bacterial families; *Pyrinomonadaceae* (MAGs 1–2), *Chitinophagaceae* (MAGs 3–4), *Cyclobacteriaceae* (MAG 5), *Hymenobacteraceae* (MAG 6), and *Rubrobacteraceae* (MAGs 7–8) (Supplemental Table 7). MAGs 9–11 had low completion (0%–4.17%) and were unclassified (Supplemental Table 7). Bacterial families from 16S rRNA gene sequencing included *Blastocatellaceae*, *Solirubrobacteraceae*, *Spirosomacea*e, *Trueperaceae*, *Hymenobacteraceae*, and *Chitinophagaceae* (Fig. 3) which were also present in dry, oligotrophic, ice-free Antarctic soils from the Shackleton Glacier region [9]. The phyla *Acidobacteriota*, *Actinobacteriota*, *Bacteroidota*, *Chloroflexi*, *Planctomycetota* are common dominant phyla in the MDVs, and have also previously been found in dry permafrost from University Valley [5]. The isolation environments of the most related sequences in the GenBank database were from dry, mountainous soil in Antarctica (such as the Sør Rondane Mountains, the Darwin Mountains, Alexander Island, and the La Gorce Mountains), other Antarctic areas (such as MDV algal mats, Mt. Erebus cave sediments), and other deserts from Norway and the Atacama (Supplemental File 1, Supplemental File 2). Most families identified in this study are known to be aerobic heterotrophs, with a few exceptions which may include facultative aerobes (*Nocardiaceae*) or facultative anaerobes (*Spirosomaceae*). The only family with organisms capable of autotrophy was *Cyanobiaceae*, represented by one ASV present in Site 3 0–10 cm.

Only one genus (*Sphingomonas*) was shared between cultivated isolates and organisms identified via 16S rRNA gene amplicon sequencing. Similar families, *Nocardiaceae*, *Hymenobacteraceae*, and *Rubrobacteraceae* were found in 16S rRNA gene amplicon, metagenome, and isolated microbiota datasets. *Arthrobacter* was the most commonly isolated genus but was not found in amplicon or metagenomic datasets. The observed difference between these datasets could be a result of the low biomass which makes both cultivation and DNA extraction difficult and may bias the two datasets toward different groups of organisms.

Fungal communities (total 193 ASVs) had higher richness than bacteria (97 vs. 20 genera) in Elephant Head permafrost (Supplemental Fig. 2), and included genera that are related to organisms found in other arid Antarctic environments; *Rhodotorula*, isolated from dry permafrost from University Valley [5, 53], *Rachicladosporium*, found in MDV endoliths [54] *Cladosporium*, from soils of Schirmacher Oasis, East Antarctica and the Heritage range of the Ellsworth Mountain system [55, 56] and *Aureobasidium pullulans* from permafrost at the Progress and Russkaya stations [57].

No archaeal sequences were identified in this study. This may be due to the low amount of DNA recovered but does align with numerous previous studies of the Dry Valleys, which identified a limited number or absence of archaeal sequences [5, 50]. Cold, dry soil from the Shackleton Glacier region, Antarctica was found to contain archaeal DNA in only 30% of samples [32].

### Functional potential of elephant head soils

**Figure 4:**
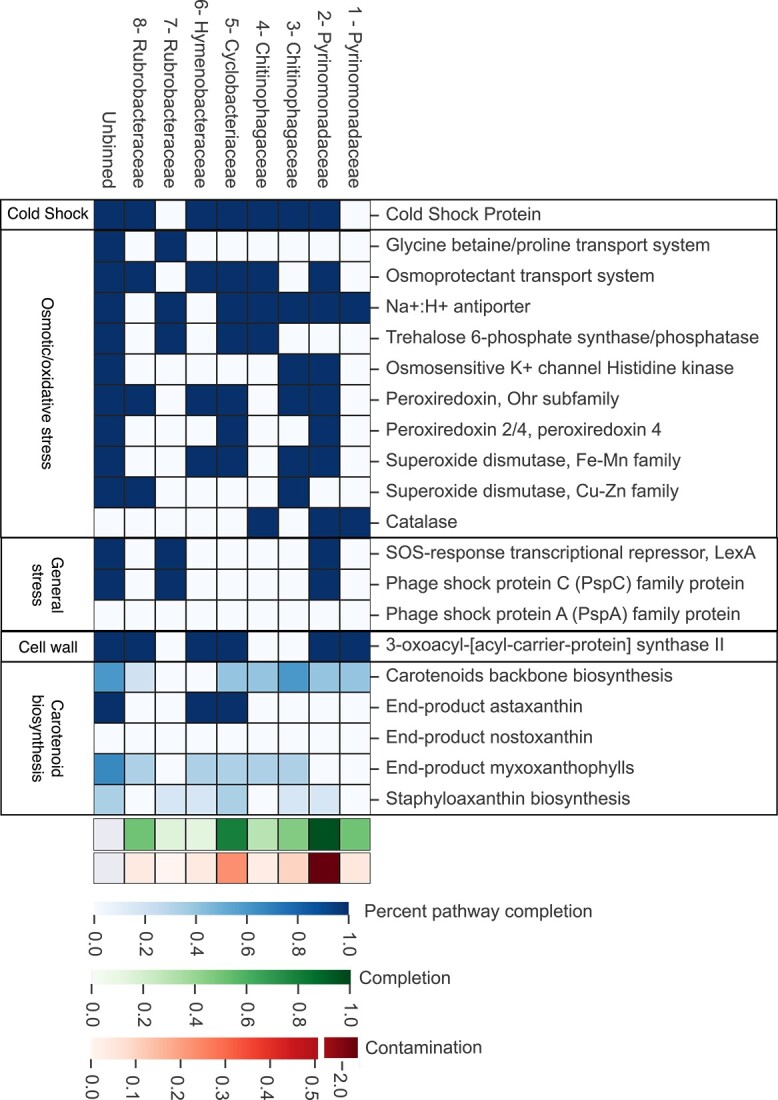
Genes associated with stress response and cold adaptation found in MAGs; blue indicates gene presence or absence except for carotenoid biosynthesis where it indicates percent pathway completion; green bar shows completion of MAGs and red bar shows contamination of MAGs based on single copy genes.

The unbinned metagenomic reads and MAGs contain genes associated with cold adaptation and stress response (Fig. 4). Genes for transport of glycine betaine and trehalose synthesis were present in the unbinned sequences, and one and three MAGs respectively (Fig. 4); however, genes for the transport of extracellular trehalose (TRET1) into the cell were absent [58]. The transport of compatible solutes and cryoprotectants including glycine betaine and trehalose across the cell membrane is important for the maintenance of cell homeostasis in desiccating and cold conditions [59]. These solutes can also lower the freezing point of intracellular water, reducing ice crystal formation which can cause cell lysis [59, 60]. Glycine betaine and trehalose are also involved in increasing membrane fluidity which is beneficial at cold temperatures [58, 61]. Coding regions for cold shock protein (CspA) were found in 5/11 MAGs as well as in the unbinned portion of the metagenome (Fig. 4). CspA helps with unfolding misfolded proteins and preventing misfolding during and after cold shock [62]. Genes encoding proteins for protection against oxidative stress were also present, including peroxiredoxin Ohr subfamily (three MAGs), superoxide dismutase (five MAGs), and catalase (three MAGs). Oxidative stress tolerance is important because more reactive oxygen species are generated at cold temperatures [62-64]. Reactive oxygen species cause cellular damage so removing them before damage occurs is important [66]. Genes for carotenoid biosynthesis were present, which is consistent with observations that most isolates were pigmented. Carotenoids are associated with protection against cold temperatures, oxidative stress, and solar radiation [43-45]. Protection against solar radiation would be important in surface soils in Antarctica, where they are exposed to intense ultraviolet radiation for large portions of the year. Pigmented Antarctic microbiota are more resistant to environmental stressors including freeze–thaw cycles and solar radiation than unpigmented bacteria [45].

**Figure 5:**
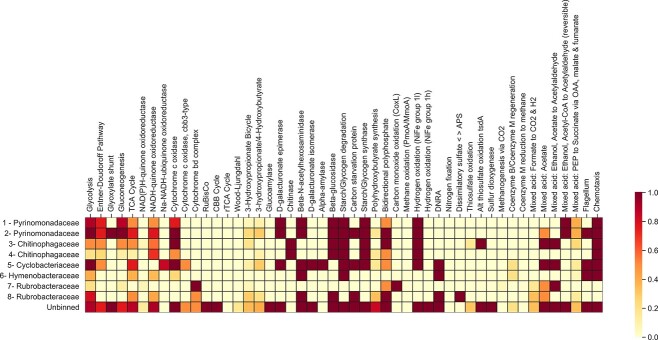
Metabolisms of MAGs; color indicates percentage pathway completeness; E-, electron acceptors; cyto, cytochromes; N, nitrogen; S, sulfur.

Genes for carbon cycling and aerobic heterotrophy were present across all MAGs (Fig. 5), and all families found are known to be aerobic heterotrophs [66-68]. Genes for the utilization of carbon sources, including starch (glucoamylase, alpha-amylase), pectin (D-galacturonate epimerase), chitin (chitinase), and cellulose (Beta-glucosidase) were also present. The ability to utilize a variety of carbon sources is important when available carbon is limited; however, it is unclear if pectin or cellulose are available carbon sources since there are no known plants or visible algae in the area. Diatoms, mosses, lichen, and cyanobacterial mats have been found in other areas of Ellsworth Land [70, 71], so it is possible they are substrates via eolian deposition. Based on ITS sequencing, fungi are present in dry permafrost from Elephant Head and may be a source of chitin. A survey of soil invertebrates in mineral soil from another area of Ellsworth Land found tardigrades, which also contain chitin [71]. The recycling of carbon from cellular material may also be important when carbon is limited. Genes for beta-*N*-acetylhexosaminidases, which are associated with peptidoglycan degradation and recycling, were present in six MAGs [72]. Photosynthesis and nitrogen fixation genes were absent.

Coding regions for starch and glycogen synthesis genes were present in five MAGs and the unbinned metagenomic reads (Fig. 5). Synthesis of starch or glycogen can be used to store energy which is important in oligotrophic conditions [73, 74]. Carbon starvation protein coding regions were also present in three MAGs and the unbinned metagenomic reads. Genes for the complete glyoxylate shunt pathway were present in one MAG (*Pyrinomonadaceae*) as well as in the unbinned reads. The glyoxylate shunt allows microorganisms to conserve carbon during the TCA cycle by avoiding the release of CO_2_. The glyoxylate shunt pathway has also been identified in a dry permafrost isolate from University Valley and is thought to help with the carbon conservation of one isolate from ~150 000-year-old permafrost [48]. The presence of the glyoxylate shunt could have reduced the amount of CO_2_ that was released and measured during radiorespiration assays.

Genes involved in trace gas metabolism were present in the metagenomes (Fig. 5). Trace gases including carbon monoxide, hydrogen, and methane are present in the atmosphere and may be a significant source of carbon or energy in nutrient limited environments [33]. The carbon monoxide oxidation gene, *coxL*, was present in one MAG which belongs to the phylum Actinobacteria. Actinobacteria have previously been identified as a phylum capable of utilizing carbon monoxide [75]. Group 1 h [NiFe]-hydrogenase was present in the unbinned metagenomic reads but was not identified in any MAGs. Group 1 l [NiFe]-hydrogenase [67] was found in the unbinned metagenomic reads, as well as three MAGs identified as the families *Chitinophagaceae*, *Pyrinomonadaceae*, and *Cyclobacteriaceae*. The potential for the oxidation of atmospheric hydrogen by Group 1 h and 1 l [NiFe]-hydrogenase has been found in multiple metagenomic studies of Antarctic deserts, often with hydrogen oxidation genes often being present across all metagenomes [33, 74-76]. Oxidation of atmospheric H_2_ by membrane bound hydrogenases produces energy through the electron transport chain and produces metabolic water which could be a significant source of hydration for cells in extremely arid environments [76]. Type 1E RuBisCo genes (associated with nonphotosynthetic carbon fixation such as trace gas metabolism) were not found in any MAGs [78].

## Conclusions

The results from Elephant Head differ from prior microbiological analyses of dry permafrost from University Valley, which described a potential natural limit to active microbial life under cold and arid conditions [4, 5]. As dry permafrost on Earth represents one of the best analogs to Martian regolith conditions, the previous findings on Earth that cold-arid conditions in the upper MDVs are prohibitive to active life have been used to inform analyses of the potential for microbial life on other cold-arid planetary bodies [78-80] including to argue that current planetary protection constraints on the planet are too stringent, as microorganisms are not likely to be able to persist [82]. Elephant Head provides the first site for comparing dry permafrost microbiology to the upper elevation MDVs and by extension the potential for microbial colonization of cold-arid Martian regolith, in particular those designated as “Special Regions” (Supplemental Discussion—Elephant Head dry permafrost as a Martian analog).

Elephant Head differs from prior findings in dry permafrost by having an order of magnitude higher total biomass (10^4^ cells/g) than what was identified in University Valley (10^3^ cells/g). The culturable microbiota from Elephant Head was also higher, and isolates demonstrated cell division at environmentally relevant temperatures under relatively clement laboratory conditions (higher nutrients and available water). Unlike University Valley dry permafrost, which only had activity in thawed samples at 5°C, microbial activity (acetate mineralization) was detected in some Elephant Head samples at subzero temperatures. Activity was at low levels that were often difficult to distinguish from abiotic background levels, potentially attributed to difficulties in detection associated with low biomass. Similar to University Valley dry permafrost, DNA yield was low in Elephant Head samples and required whole genome amplification to obtain sequence information. The microbial communities identified using genomic sequencing were similar to those found in other Antarctic and cold environments and have genomic adaptations for survival in cold, dry, oligotrophic conditions.

**Figure 6:**
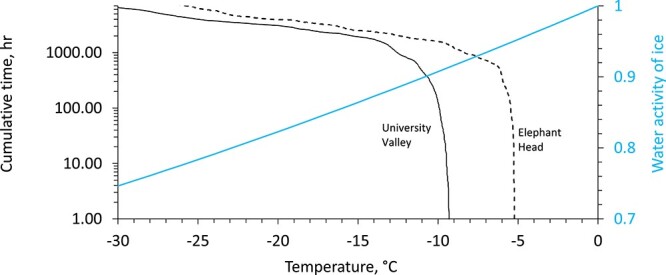
Temperature and water activity of University Valley and Elephant Head (Site 3) at the ice tables (42 cm and 49 cm respectively); the black curves show the cumulative time that is spent above a specific temperature in hours per year; the blue curve shows the water activity set by the temperature of ice; the figure is modified from Marinova *et al*. [84](Fig. 6).

Taken together, the cultivation, sequencing, and activity assay results indicate that Elephant Head soils are more habitable that those of University Valley permafrost. The difference in microbial biomass and activity could be attributed to the slightly higher average temperature and relative humidity conditions present at Elephant Head throughout the year (Fig. 6; Supplemental Table 1). The lowest temperature that microbial growth has been observed is −18°C [83], and the lowest water activity for known growth is 0.585 [82], albeit these were at relatively higher nutrient conditions than dry permafrost environments. Temperature data indicate that above the ice table, Elephant Head soils achieve temperatures above −18°C and water activity above 0.6 for ~900 h more of the year (~10% of a year) compared to University Valley. Therefore, it is possible that the difference of a few degree hours annually makes a difference for habitability and the presence of active life (Fig. 6). Regardless, our analyses at Elephant Head demonstrate that dry permafrost per se does not exclude active microbial life at subzero temperatures.

## Supplementary Material

Supplemental_Materials_ycad002

SupplementalFile_1_16S_ASVs_ycad002

SupplementalFile_2_ITS_ASVs_ycad002

## Data Availability

The metagenomic and amplicon datasets generated during and/or analyzed during the current study are available in the Genbank SRA repository under project number PRJNA922518. Sanger sequences of 16S rRNA genes of isolates are deposited in Genbank under accession numbers (OQ383637-OQ383640, OQ383889-OQ383905).
