# Supplementary material for: Active microbiota persist in dry permafrost and active layer from Elephant Head, Antarctica
Source: ISME Commun. 2024 Jan 10;4(1):ycad002. doi: 10.1093/ismeco/ycad002 (PMC10833075; doi:10.1093/ismeco/ycad002)
Supplement: Supplemental_Materials_ycad002 [file supplemental_materials_ycad002.pdf]

Supplemental Materials for Active microbiota in dry permafrost from Elephant Head, Antarctica

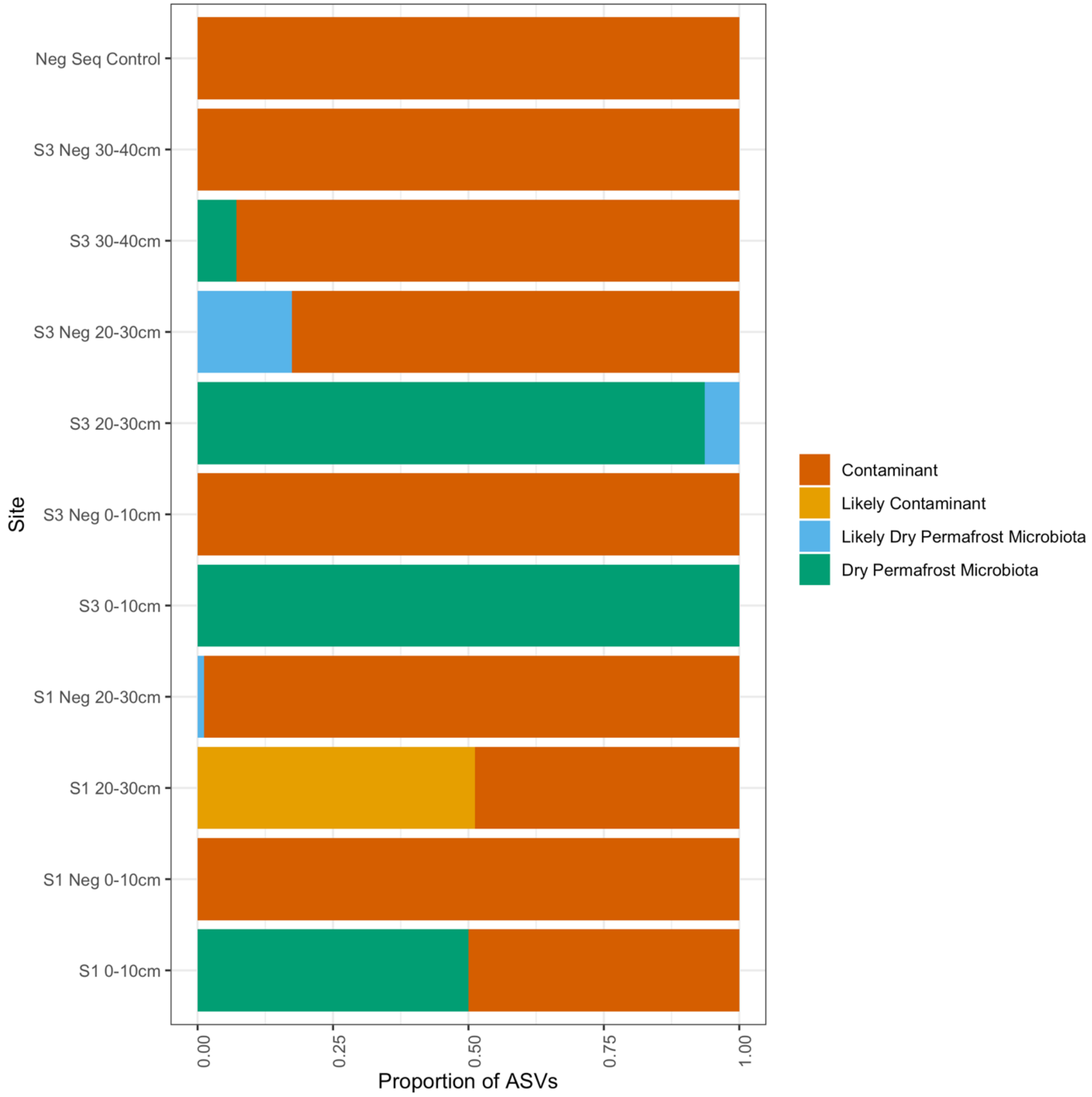

Supplemental Figure 1: Proportion of ASVs from 16S rRNA gene amplicon sequencing classified as being contaminants, likely contaminants, possibly being from dry permafrost and being from dry permafrost.

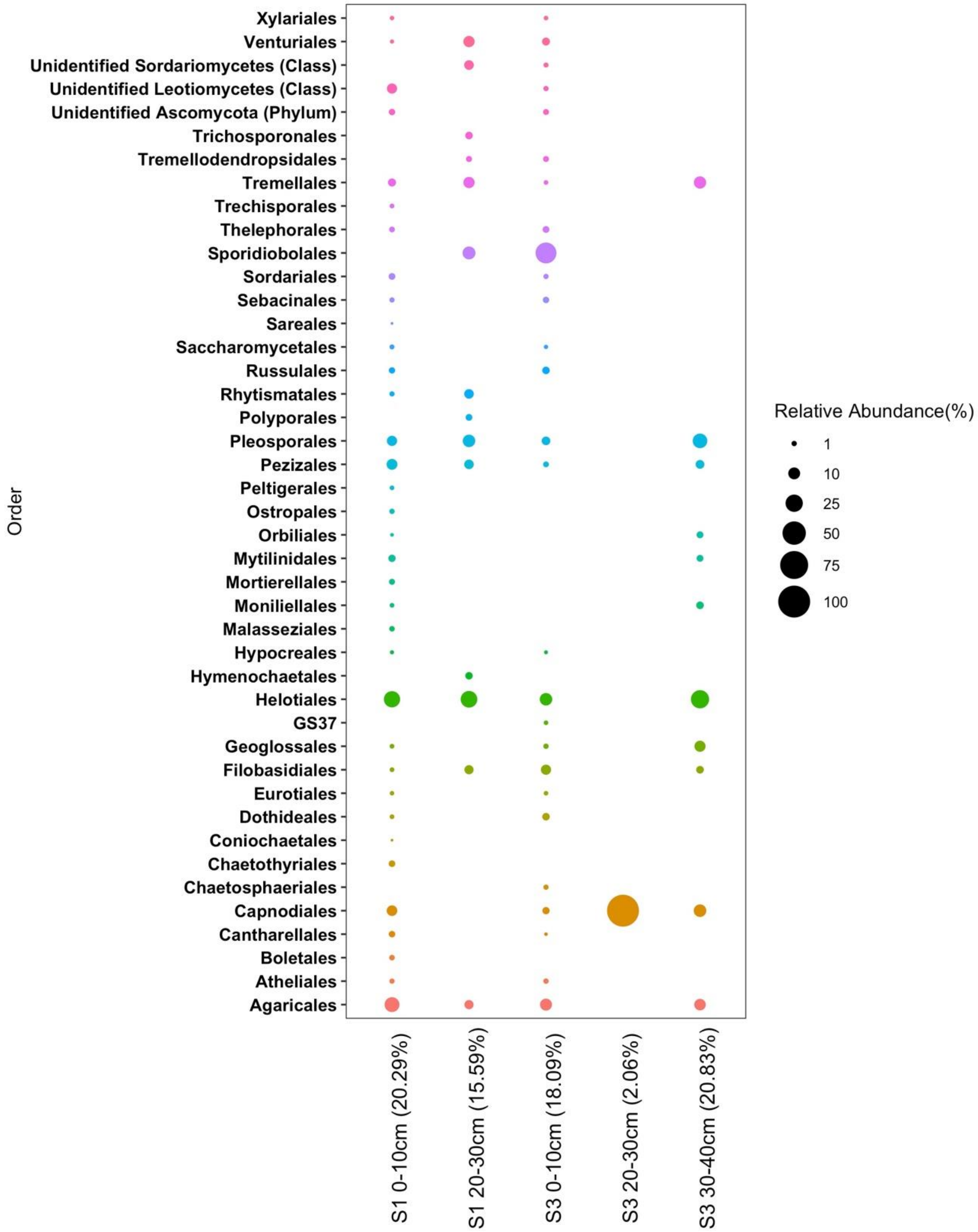

Supplemental Figure 2: Relative abundance of Fungal ASVs from ITS sequencing. Sequences present in the negative controls were removed along with any sequences not classified past the phylum level and any sequences belonging to the genus Candida. Percent indicates the percentage of sequences that were retained.

Supplemental Table 1: Comparison of temperature and relative humidity data from Elephant Head (EH) [1] and University Valley (UV)

	**EH (McKay et al 2019)**	**UV (Marinova et al 2022)**
**Air (annual)**		
T avg	-20.3°C	-23.4°C
T min	-42.3°C	-45.5°C
T max	-0.14°C	-2.8°C
RH_i avg	68.7%	45.4%
RH_i min	23.8%	21%
RH_i max	98.7%	82%
**Surface (Dec 1-Feb28)**	**3cm**	**0cm**
T max*	17.75°C	12.3°C
RH_i max*	100%	100%
**Ice Table (annual)**	**49cm**	**42cm**
T avg	-19.2°C	-24.1°C
T min	-30.3°C	-37°C
T max	-5.2°C	-9°C
RHi avg	99.7%	96.5%
RHi min	95.4%	85%
RHi max	100.7%	100%
T > -18.00°C, a_w > 0.6	3474 hours	2650 hours
T > -10°C, a_w > 0.6	1574 hours	129 hours

*Only summer data is available for EH at the surface depth; no yearly average is presented.

Supplemental Table 2: Geochemical and biomass measurements in dry permafrost and ice samples from Elephant Head, Antarctica.

	Depth	Wt. %total Carbon	Wt. % total organic carbon	δ13C vs VPDB	Organic δ13C vs VPDB	Wt % total Nitrogen	δ15N vs AIR	Cell counts[1] (cells/g)	[DNA][2](ng/uL)	Gravimetric moisture content (%)
**Site 1**										
	0-10 cm	7.31±0.12		0.91±0.2		0.007±1e-4		NE	BD	0.297
	10-20 cm	8.31±0.06		0.81±0.2		0.009±1e-5	-13±1.8	NE	NE	0.181
	20-30 cm	5.33±0.11		0.45±0.2		0.001±3e-4	-9±1.2	NE	-	0.425
	Ice ()							NE	NE	
**Site 3**										
	0-10 cm	10.52±0.33	0.07*	0.98±0.2	-28.25	0.004±4e-4		3.31 x 10^4	0.0662	0.076
	10-20 cm	11.01±0.12		1.4±0.2		0.005±4e-5		NE	NE	0.004
	20-30 cm	10.30±0.25		1.01±0.2		0.011±5±-4	-15±2.0	NE	-	1.21
	30-40 cm	11.11±0.03		0.91±0.2		0.01±2e-4	-17.9±0.80	NE	BD	1.04
	Ice ()							NE	NE	
**Arctic**	0-5 cm							1.12 x 10^8	-	

[1] Cell counts with SYTO 9 green fluorescent nucleic acid stain. [2][DNA] is the concentration of DNA following extraction measured on a Qubit. BD (Below Detection) - not detectable on the Qubit, - indicates that raw DNA concentration was not measured to preserve sample for MDA attempts. NE (No Extraction) - indicates that no microscopy or DNA extraction was performed due to not having enough sample. VPDB - Vienna Pee Dee Belemnite. * These measurements were low, and outside of the calibration range of the instrument.

Supplemental Table 3: Ion measurements in dry permafrost and ice samples from Elephant Head, Antarctica.

	Depth	Replicates measured	Chloride (mg/kg)	Chloride Std. dev	Nitrate	Nitrate std dev	Sulfate	Sulfate std dev
**Site 1**								
	0-10 cm	2	184	0.01	132	0.01	428	0.07
	10-20 cm	2	194	0.03	137	0.01	281	0.05
	20-30 cm	2	271	0.02	191	0.01	240	0.02
	Ice	2	5	0.00	1	0.00	3	0.00
**Site 3**								
	0-10 cm	10	11	0.05	10	0.04	71	0.06
	10-20 cm	2	69	0.00	69	0.00	183	0.01
	20-30 cm	2	100	0.00	108	0.01	293	0.00
	30-40 cm	4	283	0.01	278	0.00	255	0.01
	Ice	2	5	0.00	2	0.00	3	0.01

Std. dev. Standard deviation of measurements

Supplemental Table 4: Average cumulative mineralization of radiorespiration assays after 318 days.

Site	Depth	Sample[1]	Negative Control[2]	n[3]	p-value[4]	Significance[5]
			**5°C**			
Site 1	0-10	0.99504417	0.78288896	3	0.00194	**+
Site 1	10-30	1.10432714	1.20686749	3	0.19071	
Site 1	Ice	0.48090133	0.53737521	2	0.06014	
Site 3	0-10	1.06701211	1.18649986	3	0.22085	
Site 3	10-30	0.83753569	1.24597473	3	0.04054	*
Site 3	30-40	0.86770283	1.05494176	3	0.03982	*
Site 3	Ice	0.60058647	0.57328407	3	0.00266	**+
Arctic	0-5	62.1170419	0.58902269	3	2.68E-05	****+
			**0°C**			
Site 1	0-10	1.20927457	1.33998024	3	0.01076	**
Site 1	10-30	1.08159785	0.92770174	3	0.02766	*+
Site 1	Ice	0.8534944	0.66202823	3	0.00058	***+
Site 3	0-10	0.61103754	1.33998024	3	0.00961	**
Site 3	10-30	1.0792292	0.8675002	3	0.00830	**+
Site 3	30-40	0.85603424	0.84073581	3	0.14110	
Site 3	Ice	0.99038023	0.84176293	3	0.01528	*+
Arctic	0-5	53.5781396	0.71090005	3	0.00026	***+
			**-5°C**			
Site 1	0-10	1.10164756	0.68703534	2	0.00900	**+
Site 1	10-30	1.07816714	0.92770174	3	0.00249	**+
Site 1	Ice	0.99038023	0.65759137	3	0.34851	
Site 3	0-10	1.11518871	1.26861668	3	0.02946	*
Site 3	10-30	0.89534411	0.86494639	3	0.33571	
Site 3	30-40	0.74249957	0.65306717	3	0.05916	
Site 3	Ice	0.74878803	1.02123561	3	0.02943	*
Arctic	0-5	8.29257275	0.51279256	2	0.01359	*+

[1]Final cumulative mineralization of samples after 318 days (average of n where [3]n=number of replicates). [2]Final cumulative mineralization of negative controls after 318 days (average of n). [4]p-value is a result of a paired one tailed t-test. [5]Significance is based off p-value; *p<0.05, **p<0.01, ***p<0.001, ****p<0.0001. + indicates that the sample was significantly higher than the negative control.

Supplemental Table 5: Number of 16S rRNA gene sequence reads through the DADA2 pipeline and manual curation steps. Number indicates the amount of reads remaining following that step.

	Input	Filtered	DenoisedF	DenoisedR	Merged	Nonchim	Rem_contam	Rem_likely_contam	%_Reads_Kept
S1-010	129	98	90	90	90	90	57	57	44.19
S1-010N	57	41	34	34	23	21	0	0	0.00
S1-2030	100993	79807	79755	79766	79728	63112	1079	0	0.00
S1-2030N	11641	9648	9630	9629	9588	7335	6	6	0.05
S3-010	7659	6372	6300	6129	5887	3460	3460	3460	45.18
S3-010N	7444	5863	5811	5826	5790	4510	0	0	0.00
S3-2030	66485	55629	55547	55605	55543	39246	39246	39246	59.03
S3-2030N	720	602	569	559	499	457	71	71	9.86
S3-3040	14945	12367	12352	12358	12350	9695	48	48	0.32
S3-3040N	7974	6591	6582	6577	6577	5065	0	0	0.00
Seq Neg	24	15	4	4	4	4	0	0	0.00
Total	218071	177033	176674	176577	176079	132995	43967	42888	19.67

Abbreviations: N indicates negative control. denoisedF – denoised forward, denoisedR – denoised R, nonchim – non-chimeras, rem_contam – removal of ASVs categorized as contaminants, rem_likely_contam – removal of ASVs categorized as likely contaminants.

Supplemental Table 6: Number is ITS sequence reads through the DADA2 pipeline and manual curation steps. Number indicates the amount of reads remaining following that step.

	Input	Filtered	DenoisedF	DenoisedR	Merged	Nonchim	Rem_neg	Manual_removal	%_Reads_Kept
S1-010	4278	2129	2022	2002	1866	1866	879	868	20.29
S1-010N	4168	1964	1939	1941	1831	1831	0	0	0.00
S1-2030	898	328	300	285	268	268	145	140	15.59
S1-2030N	2627	735	728	728	728	728	0	0	0.00
S3-010	5324	2825	2789	2772	2682	2682	1018	963	18.09
S3-010N	134	35	25	19	19	19	0	0	0.00
S3-2030	97	5	2	2	2	2	2	2	2.06
S3-2030N	270	71	58	58	36	36	0	0	0.00
S3-3040	629	286	233	226	199	199	139	131	20.83
S3-3040N	80	12	4	4	4	4	0	0	0.00
Seq Neg	70	14	1	1	0	0	0	0	0.00
Total	18575	8404	8101	8038	7635	7635	2183	2104	11.33

Abbreviations: denoisedF – denoised forward, denoisedR – denoised R, nonchim – non-chimeras, rem_neg – removed sequences present in the negative controls, manual_removal – removed sequences that were not identified past the phylum level and those identified as the genus *Candida*.

Supplemental Table 7: Metagenome assembled genome information

Bin #	Classification (Family)	Completion (%)	Contamination (%)	Length	# Contigs	N50	GC content	Description of Manual refinement
1	Pyrinomonadaceae	51.03	5.17	2073613	615	3819	48.85	NA
2	Pyrinomonadaceae	96.39	206.55	13327405	4044	3624	45.11	NA
3	Chitinophagaceae	46.38	10.34	3950533	1338	3082	37.60	Manual refinement based on sequence composition + differential coverage showed two trees. Inspecting the redundant single copy genes showed that many of the duplicate genes had one copy on each of the two branches so the split was made into two bins (Bins 3 and 4)
4	Chitinophagaceae	30.28	3.45	2446851	672	4147	37.65	
5	Cyclobacteriaceae	80.12	22.65	4802251	918	6474	41.72	NA
6	Hymenobacteraceae	12.5	4.17	1402443	324	4864	46.66	NA
7	Rubrobacteraceae	14.55	0.44	984002	351	2934	64.51	Manual refinement based on sequence composition + differential coverage and presence of single copy genes split one bin into bins 7 and 8.
8	Rubrobacteraceae	50.96	4.39	2154148	571	4322	63.10	
9	Unclassified	4.17	0	475464	167	3089	36.98	NA
10	Unclassified	4.17	0	264181	68	4658	60.90	NA
11	Unclassified	0	0	317046	96	3498	59.09	NA
Unbinned	NA	NA	NA	26234594	13780	1866	48.55	NA

Supplemental Discussion

1. **Elephant Head dry permafrost as a Martian analog**

Water activity (a_w) is used to describe the amount of water available for microbial use and decreases as water freezes or as the amount of solutes increases. Dry permafrost is characterized by low water activity and low temperatures, which is also used to define Special Regions on Mars where life could exist. Special Regions are defined by [2] as follows: "Special Regions on Mars continue to be best determined by locations where both of the parameters (without margins added) of temperature (above 255K, -18°C) and water activity (aw > 0.60) are attained." At Elephant Head from December 1, 2017 to November 30, 2018 there were 3474 hours at the ice table when temperatures exceeded -18°C ($a_w > 0.6$) which accounts for approximately 40% of the year. This is ~10% higher than at University Valley in the McMurdo Dry Valleys (T > -18°C, aw > 0.6 = 2560 hours) [3]. Figure 6 of the main text shows the cumulative time spent above a specified temperature for Elephant Head compared to University Valley. Water activity never goes below the 0.6 cut-off for Mars Special Regions for any of the temperatures recorded at either dry permafrost sites, thus both sites would be considered Special Regions [3].

Supplemental Methods

Manual Contaminant screening of amplicon sequence data

For 16S rRNA gene data, ASVs were ranked as either contaminants, likely contaminants, likely dry permafrost microbiota or dry permafrost microbiota (Supplemental Figure 1) according to the following criteria: An ASV was categorized as a "contaminant" if it was present only in the negative controls, or if it was found in the negative controls and the samples and did not have a closest GenBank environment match to Antarctica or another cold environment. An ASV was categorized as a "likely contaminant" if it was found in the samples only (no reads in the negative controls) and had a closest GenBank environment match to skin. "Likely dry permafrost microbiota" were ASVs that has closest GenBank matches to Antarctica or other cold environments and were found in both the samples and negative controls. "Dry permafrost microbiota" were ASVs were those present only in the samples and were not excluded based on any of the previous criteria. Any sequences categorized as contaminants or likely contaminants were removed from further analysis. After contaminant removal, ~20% of 16S rRNA gene reads remained (

Supplemental Table **5**).

Only four ASVs were categorized as "likely dry permafrost microbiota," all from the genus Delftia. These ASVs were present in site 3 (20-30 cm) samples (4013 reads) and two DNA extraction negative controls (Site 1 and 3 20-30 cm extraction control; 77 reads). Due to the differential abundance, the presence of the ASVs in the negative controls may have been due to barcode switching as follows. During Illumina MiSeq sequencing, samples are barcoded with short sequences of bases to allow for multiplexing of samples. These barcodes may switch from their original amplicon to another amplicon while passing through the flow cell. This is known as barcode switching and results in that amplicon being assigned to a different sample than it was originally from [4]. While normally a minor contribution to a total community, the effects are more pronounced in extreme low biomass samples.

For ITS sequences any sequences present in the negative controls were removed. Sequences that were not identified to the phylum level and were found to have closest GenBank matches to algae were removed (5 ASVs). Manual curation based on environment match was not possible due to the number of different ASVs. Common skin fungi belonging to the genus *Candida* were removed (3 ASVs). After contaminant removal ~11% of ITS sequences remained (Supplemental Table 6).

Ion Chromatography

Permafrost soil samples were crushed and homogenized with a combusted mortar and pestle and extracted in milli-Q water at a ratio of 1:1 by mass. Extracts were shaken for 30 s to mix by vortex and centrifuged at 4500 xg for 10 min at 4°C. The supernatant was filtered through a 0.2 μm filter (PES, Sterlitech) and frozen until IC analysis. Remaining soil was dried

overnight in an incubator oven (72°C) to record dry weight. Soil nutrient values are presented as mass per kg dry soil. Ice samples were prepared by thawing overnight at 4°C. Thawed samples were centrifuged at 1000 xg for 10 min at 4°C. The supernatant was filtered through a 0.2 μm filter and stored frozen until analysis. Ice nutrient values are presented as mass per kg water. All extracts were then diluted 1:10 before IC analysis.

Methods for IC are from [5] with relevant details as follows. Major anions ions were quantified using a Dionex DX-600 ion chromatography system operated by Chromeleon software (version 6.8). The anion system employs a potassium hydroxide eluent generator, a carbonate removal device, and an AS18 (4x250mm) analytical column (Thermo catalog number: 060549) coupled with an AG18 (4x50mm) guard column (Thermo catalog number: 060551). The hydroxide concentration of the eluent is held isocratically at 5 mM for 5 minutes, followed by a non-linear (Chromeleon curve 8 hydroxide concentration gradient to 55 mM applied over 31 minutes, after which the column is re-equilibrated at 5 mM hydroxide for 10 minutes before the next sample injection. The eluent flow rate is held constant at 1.0 mL/minute. The system is plumbed with an external source of deionized water for suppressor regeneration to improve the signal-to-noise ratio of the analyses and suppressor currents is 137 mA. Samples were delivered to the instrument from 5 mL vials via AS-40 autosamplers (2 injections per vial) onto 100 μL sample loops. Quantification is achieved externally via calibration curves constructed from a series of dilutions of mixed-ion standards (Environmental Express, Charleston, SC, USA). Quantification accuracy is verified daily by analysis of an independent mixed ion standard (Thermo Scientific, Waltham, MA, USA). Data is presented as the mean and standard deviation of all injections. Uncertainties in reported ion concentrations are estimated to be ± 5%.

Elemental Analysis

Permafrost soil samples were crushed and homogenized with a combusted mortar and pestle. For carbon analyses, 10 mg soil was weighed into tin cups in triplicate using a microbalance. This was sufficient sample for total carbon content and isotope composition, but insufficient for total nitrogen content and isotope composition. For nitrogen analyses, 30 mg soil was weighed into tin cups in triplicate using a microbalance. However, this was still not sufficient to determine isotope composition for all samples and delta values are omitted for these samples.

One sample with excess material was weighed into silver cups and acid digested using methods described in [6] to determine organic carbon abundance and isotope composition. Briefly, 20 µL of MilliQ water was added to wet the sample prior to acid digestion. 50 µL of 6M HCl was added in 10 µL increments until visible effervescence ceased and samples were dried overnight (72°C) before weighing.

Analytical Glycine standards were used as calibration. In-house glycine standards were characterized using USGS40 and USGS41a reference material. Glycine Mid and NIST2710 were analyzed as QC of scale calibration. Stable carbon and nitrogen isotopic compositions were determined using Thermo Delta Plus Advantage continuous flow isotope ratio mass spectrometer coupled to a Costech ECS 4010 elemental analyzer at Arizona State University. Samples were combusted at 1020°C in a reactor packed with chromium oxide and silvered cobaltous oxide. Following combustion, oxides were removed in a reduction reactor (reduced copper at 650°C). N_2 and CO_2 were separated on a GC column at 55°C before entering the IRMS.

Stable carbon isotopic compositions are expressed as delta values relative to VPDB (Vienna Pee Dee Belemnite) on a scale normalized such that the $\delta^{13}C$ values of NBS 19 calcium

carbonate and L-SVEC lithium carbonate are +1.95 ‰ and –46.6 ‰, respectively. Stable nitrogen isotopic compositions are expressed relative to atmospheric nitrogen, which is isotopically homogeneous. The long-term standard deviation is 0.2 ‰ for $\delta^{13}C$ and 0.3 ‰ for $\delta^{15}N$.

Fatty Acid Analysis

Lipid extractions were performed on soil samples from sites 1 and 3 utilizing extraction protocols developed specifically for organically lean soils [7]. In short, samples were extracted using a modified Bligh and Dyer technique: extraction of ~110 g of soil with of 40 mL of water, 100 mL of methanol, and 50 mL of dichloromethane three times using stirring and ultrasonic sonication, filtration, concentration of extract, methanolysis to generate fatty acid methyl esters (FAMEs), and injection onto an Agilent 8890 GC-MS [7]. The final volume was 100 µL, which contained 25% of the derivatized total lipid extracts, and 1/100 µL was injected into the GC-MS.

FAMEs were detected only at site 3, however they were found low in abundance, less than the limit of quantitation of the GC-MS of ~30 ng $C_{16:0}$ fatty acid /µL, but slightly above the measured limit of detection of ~5 ng $C_{16:0}$ fatty acid /µL. These concentrations of approximately 10 ng FAME/g of soil detected at site 3 in Elephant Head are similar or below the "organically leanest" samples analyzed in the hyper-arid core of the Atacama Desert [7, 8]. Only six FAMEs were identified at site 3 including two normal, straight chain fatty acids, two monounsaturates, and two fatty acids containing a methyl branch. Because of the low counts on the diagnostic molecular "M^{+}" ion, the chain length of the FAMES could not be determined from the resulting GC-MS data. Further work is being conducted to extract and characterize lipids from these

extremely biomass-poor soils. Our results indicate that future life detection efforts on Mars will likely require instruments to have an exceptionally low limit of detection.

Direct microscopic cell counts

Soil (1 g) was added to 1 mL of 0.85% NaCl. The suspension was vortexed for five minutes on low speed to suspend cells in the liquid. The liquid was transferred to a new 2 mL tube by pipette and centrifuged for 15 seconds at 3000 rpm to pellet sediment. The supernatant was transferred to a clean tube and 1 μL of SYTO 9 green fluorescent nucleic acid stain (Invitrogen) was added. Cells were stained for 30 minutes in the dark and then filtered through a 0.2 μm black polycarbonate filter. The filter was rinsed with 10 mL of 0.85% NaCl to remove excess stain. The filter was placed onto a microscope slide and a drop of PromoFluor Antifade Reagent (PromoKine) was added before placing the coverslip. Cells were counted on a LEICA DM 5000 fluorescence microscope at 1000x (oil immersion). 10 random areas were counted and averaged to calculate the number of cells per gram of soil.

Supplemental References

1. McKay CP, Balaban E, Abrahams S, Lewis N. Dry permafrost over ice-cemented ground at Elephant Head, Ellsworth Land, Antarctica. *Antarct Sci* 2019; **31**: 263–270.
2. Rummel JD, Beaty DW, Jones MA, Bakermans C, Barlow NG, Boston PJ, et al. A New Analysis of Mars '"Special Regions"': Findings of the Second MEPAG Special Regions Science Analysis Group (SR-SAG2). *Astrobiology* 2014; **14**: 887–968.
3. Marinova MM, McKay CP, Heldmann JL, Goordial J, Lacelle D, Pollard WH, et al. Climate and energy balance of the ground in University Valley, Antarctica. *Antarct Sci* 2022; **34**: 144–171.
4. D'Amore R, Ijaz UZ, Schirmer M, Kenny JG, Gregory R, Darby AC, et al. A comprehensive benchmarking study of protocols and sequencing platforms for 16S rRNA community profiling. *BMC Genomics* 2016; **17**: 55.
5. Fecteau KM, Boyd ES, Lindsay MR, Amenabar MJ, Robinson KJ, Debes RV, et al. Cyanobacteria and Algae Meet at the Limits of Their Habitat Ranges in Moderately Acidic Hot Springs. *J Geophys Res Biogeosci* 2022; **127**.
6. Brodie CR, Leng MJ, Casford JSL, Kendrick CP, Lloyd JM, Yongqiang Z, et al. Evidence for bias in C and N concentrations and δ13C composition of terrestrial and aquatic organic materials due to pre-analysis acid preparation methods. *Chem Geol* 2011; **282**: 67–83.
7. Wilhelm MB, Davila AF, Eigenbrode JL, Parenteau MN, Jahnke LL, Liu X-L, et al. Xeropreservation of functionalized lipid biomarkers in hyperarid soils in the Atacama Desert. *Org Geochem* 2017; **103**: 97–104.
8. Wilhelm MB, Davila AF, Parenteau MN, Jahnke LL, Abate M, Cooper G, et al. Constraints on the Metabolic Activity of Microorganisms in Atacama Surface Soils Inferred from Refractory Biomarkers: Implications for Martian Habitability and Biomarker Detection. *Astrobiology* 2018; **18**: 955–966.
